# Multi metabolomics-based analysis of application of *Astragalus membranaceus* in the treatment of hyperuricemia

**DOI:** 10.3389/fphar.2022.948939

**Published:** 2022-07-22

**Authors:** Wenwen Zhang, Yifang Cui, Jiayu Zhang

**Affiliations:** ^1^ The School of Pharmacy, Binzhou Medical University, Yantai, China; ^2^ The School of Pharmacy, Shandong University of Traditional Chinese Medicine, Jinan, China

**Keywords:** hyperuricemia, metabolomics, *Astragalus* membranaceus, biomarker, metabolic pathways

## Abstract

Hyperuricemia (HUA) is a common metabolic disease that is an independent risk factor for comorbidities such as hypertension, chronic kidney disease, and coronary artery disease. The prevalence of HUA has increased over the last several decades with improved living standards and increased lifespans. Metabolites are considered the most direct reflection of individual physiological and pathological conditions, and represent attractive candidates to provide deep insights into disease phenotypes. Metabolomics, a technique used to profile metabolites in biofluids and tissues, is a powerful tool for identification of novel biomarkers, and can be used to provide valuable insights into the etiopathogenesis of metabolic diseases and to evaluate the efficacy of drugs. In this study, multi metabolomics-based analysis of the blood, urine, and feces of rats with HUA showed that HUA significantly altered metabolite profiles. *Astragalus membranaceus* (AM) and benbromomalone significantly mitigated these changes in blood and feces, but not in urine. Some crucial metabolic pathways including lipid metabolism, lipid signaling, hormones synthesis, unsaturated fatty acid (UFAs) absorption, and tryptophan metabolism, were seriously disrupted in HUA rats. In addition, AM administration exerted better treatment effects on HUA than benbromomalone. Furthermore, additional supplementation with UFAs and tryptophan may also induce therapeutic effects against HUA.

## Introduction

Metabolomics is a powerful emerging technique used to investigate global changes in numerous metabolites within organisms under different conditions or environments (Bujak et al., 2015; Johnson et al., 2016). Endogenous metabolites, pharmaceutical metabolites, and products of co-metabolism between the host and gut microbiota can be evaluated using metabolomics (Johnson et al., 2016). Host pathological states can be reflected by minor changes in endogenous and exogenous metabolites. Therefore, metabolomics has become an essential tool for investigation of metabolic processes, identification of potential biomarkers, and characterization of metabolic reprogramming in various diseases (Rangel-Huerta et al., 2019; Deng et al., 2020).

Hyperuricemia (HUA) can be caused by overproduction of urate, or, far more commonly, by inefficient excretion by the kidneys (Su et al., 2020). HUA is clinically defined as the serum UA level ≥7 mg/dl in men and postmenopausal women and ≥6 mg/dl in premenopausal women, causing various diseases, such as gout and urinary stones (Mallatr et al., 2016; Wang et al., 2018). In addition, HUA is considered to promote development or progression of cardiovascular and renal diseases (Wu et al., 2017; Cote et al., 2020; Kobalava and Troitskaya 2020). The worldwide incidence and prevalence of HUA appear to be increasing for the changes in lifestyle and the increasing population of older people (Luk and Simkin 2005). Lowering urate chemical drugs such as benbromomalone, allopurinol or febuxostat were widely used in clinical treatment for HUA. However, some reports show that chronic administration of these chemical drugs can have toxic side effects (Rey et al., 2019; Ye et al., 2019). Due to the limitations of currently available drugs, the development of new ones with more potency, different pharmacological mechanisms, and less toxicity is an active field of research.

*Astragalus membranaceus* (AM), named Huangqi in Chinese, is a fundamental herb in traditional Chinese medicine (TCM) that is used for the treatment of kidney disease in China and East Asia (Hei et al., 2005; Fu et al., 2014; Shahzad et al., 2016). Rat models have been developed to help dissect the regulatory mechanisms of human HUA. Chemical inhibition of uricase is an effective approach to generate rat with increased serum urate concentrations (Lu et al., 2019). In this study, potassium oxonate, a selectively competitive uricase inhibitor, was used to induce HUA of the rats.

We previously found that *Astragalus membranaceus* ultrafine powder (AMUP) alleviated HUA in rats with greater efficacy than benbromomalone. To further characterize changes in metabolites in rats with HUA before and after drug administration, we performed metabolomic analysis on multiple biological matrices, including plasma, urine, and feces, in rats with HUA and rats with HUA rats treated with AMUP and benbromomalone.

## Method and material

### *Astragalus membranaceus* ultrafine powder

The wild dried root of *Astragalus membranaceus* Bunge [syn.: *Astragalus membranaceus* (Fisch.) Bunge] was obtained from Shanxi Hunyuan, Wansheng *Astragalus* Development Co., Ltd. The AM roots were cut into segments ∼2–3 cm long and baked in an oven at 60°C for 4 h. After drying, the segments were crushed using an ultrafine grinder and then screened through a 400-mesh sieve to obtain AMUP.

### Animals, drug administration, biological sample collection and preparation

Pathogen-free male Sprague–Dawley rats (weight 180–220 g) were obtained from Jinan Pengyue Experimental Animal Breeding Company [license number: SCXK (LU) 20190003]. Animal experiments were conducted following the internationally accepted principles for laboratory animal use and care as found in the European Community guidelines (EEC Directive of 1986; 86/609/EEC). The rats were raised in an animal quarter at a temperature of 22 ± 2°C with 12 h light/12 h dark cycle and 50 ± 10% humidity.

The rats were randomly divided into a control group (C group), HUA model group (M group), positive drug (benzbromarone) group (B group), and low-dose AMUP group (L group), and high-dose AMUP group (H group). The rats were then labelled and weighed. Before the day of administration, the rats were fasted for 12 h but were allowed access to water. The rats in the five groups were given the following intervention measures: the C group was not assigned any intervention; the other four groups received intragastric administration of 300 mg kg^−1^ oxonic acid potassium at 8 am and fed 10% fructose water. After that, B group, L group and H group received intragastric administration twice daily (9 am and 4:00 pm) for 24 days of 20 mg kg^−1^ benzbromarone solution, 1.5 g kg^−1^ AMUP, or 3 g kg^−1^ AMUP, respectively. All drugs were dispersed in 10 ml of distilled water. The C group was gavaged with the same volume of water. All the animals in the five groups had regular forage.

Blood samples were collected by a retro-orbital bleed into heparinized tubes and immediately centrifuged at 13,00 g for 10 min to obtain plasma. All rats were put into metabolic cages for urine and feces sample collection. All samples were stored at −80°C for later analysis.

### Ultra performance liquid chromatography-mass spectrometer

Plasma, urine and stool samples for Ultra performance liquid chromatography-mass spectrometer (UPLC–MS) analysis were prepared according to a previous report with minor modifications (Wikoff et al., 2009; Liu et al., 2017). Each plasma sample (1 ml) was precipitated with 3 ml of methanol. After vortexing for 1 min, the sample was centrifuged at 3000 rpm for 10 min. The organic layer was then transferred to a fresh tube and evaporated to dryness. The residue was reconstituted in 1 ml of methanol and centrifuged at 13,000 rpm for 10 min 2 µl of the supernatant was injected into the UPLC–MS system for analysis. Urine and stool samples were thawed at room temperature. One volume of each urine sample was added to three volumes of methanol, and stool samples were extracted twice with 6 times the amount of methanol. Samples were centrifuged at 13,000 g for 10 min and filtered through a 0.22 µm membrane filter. Finally, 2 µl of the supernatant was injected into the UPLC–MS system for analysis.

### Data analysis

The pretreated data were uploaded into SIMCA-P (version 14.1, Umetrics, Umea, Sweden) for multivariate data analysis (Wiley et al., 2021). Orthogonal partial least-squares discriminant analysis (OPLS-DA) is a statistical method of supervised discriminant analysis. The grouping variables are included in the modeling for pair-wise analysis to make group differences and differences between two metabolic profiles clearer, thus enabling the screening of different metabolites. Twenty recalculated permutations were used to evaluate the validity of the model. The variable importance in the projection (VIP) value of each variable in the OPLS-DA model was calculated to indicate its contribution to the classification. Metabolites with a VIP value > 1 as well as |*p* (corr)| > 0.3 were further evaluated using the Mann–Whitney U test to determine the significance of each metabolite, with *p* < 0.05 considered statistically significant. A heatmap plot and bar plot were constructed using the R package program. Metabo Analyst 5.0 database (http://www.MetaboAnalyst.ca/) was used for the pathway analysis.

## Results and discussion

### Hyperuricemia altered the metabolite profiles of plasma, urine, and feces in rat

**FIGURE 1:**
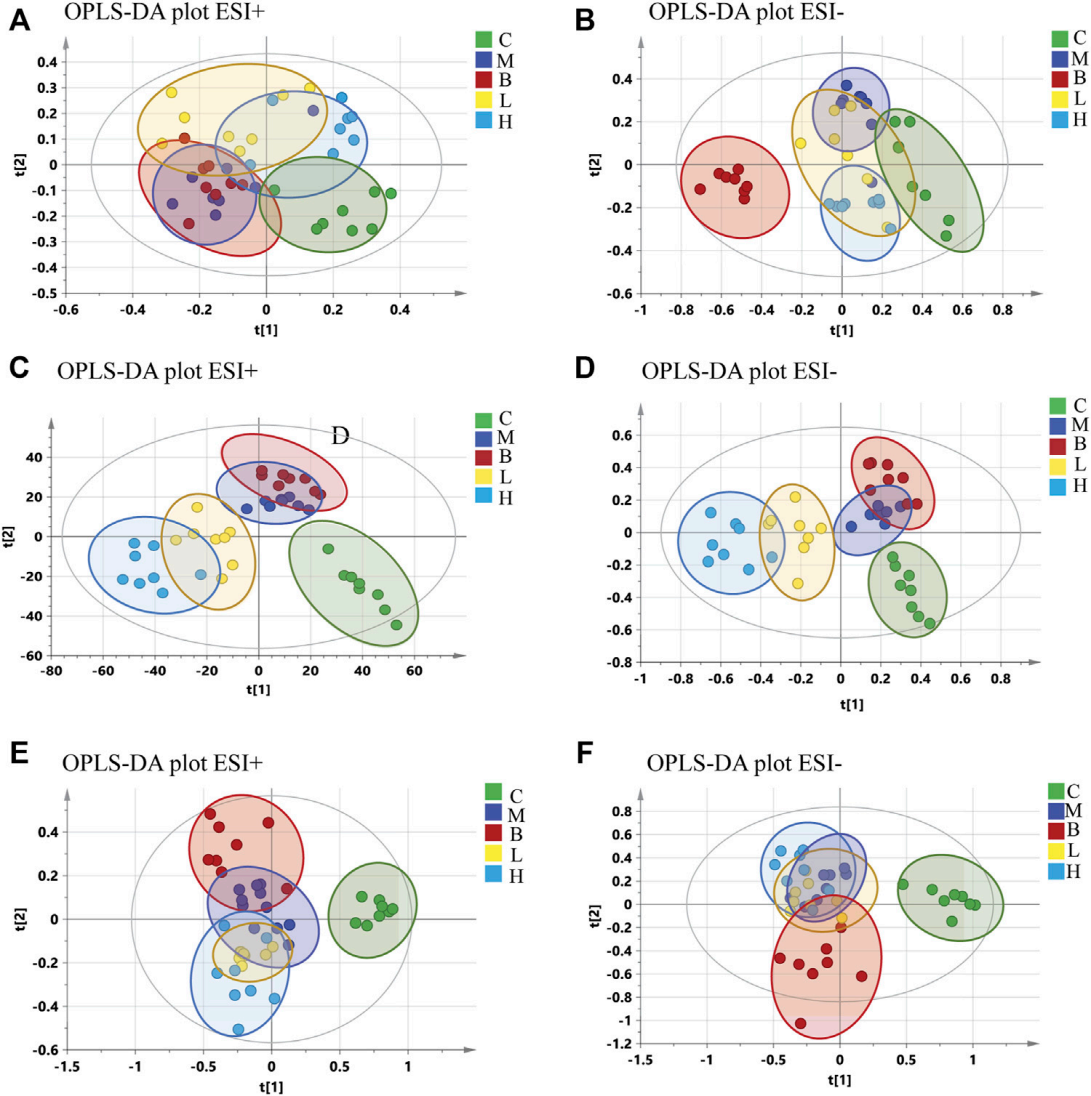
OPLS-DA of all samples in control group **(C)**, HUA model group (M), positive drug (benzbromarone) group (B), low-dose AMUP group (L), and high-dose AMUP group (H). **(A)** Score scatter plot of the blood samples in positive mode. **(B)** Score scatter plot of the blood samples in negative mode. **(C)** Score scatter plot of the feces samples in positive mode. **(D)** Score scatter plot of the feces samples in negative mode. **(E)** Score scatter plot of the urine samples in positive mode. **(F)** Score scatter plot of the urine samples in negative mode.

Partial least-squares discriminant analysis (PLS-DA) models were established to observe the magnitude of differences among the groups. The closeness of points in the model indicates metabolic composition similarity. Figures 1A,B showed that groups C and M were differentiated, especially in positive mode (Figure 1A). Group H was more similar to group C than other groups, which suggested that a high dose of AMUP could be more effective in regulating the migratory metabolome. As shown in Supplementary Table S1, 43 metabolites were differentially abundant in the plasma of HUA rats compared with normal rats. Those 43 metabolites were enrichment in five pathways including fatty acid degradation, pyrimidine metabolism, tryptophan metabolism, steroid hormone biosynthesis and glycerophospholipid metabolism (Table 1). The metabolites profiles of feces in HUA rats and normal rats showed great difference (Figures 1C,D). Seventeen metabolites enriched in linolenic acid metabolism, biosynthesis of unsaturated fatty acids and steroid hormone biosynthesis and primary bile acid biosynthesis were differentially abundant in the feces of HUA rats and normal rats (Table 1 and Supplementary Table S1). In Figures 1C,D, group B overlapped with group M, which suggested that benbromomalone normalized the metabolome to a lesser degree than AMUP. Figures 1E,F show all urine samples, and groups M and C were significantly differentiated in positive or negative mode. Pathway analysis showed that 30 differentially abundant metabolites in urine were enrichment in phenylalanine metabolism, riboflavin metabolism, vitamin B6 metabolism (Table 1 and Supplementary Table S1). The benbromomalone and AMUP groups were further from group C, which indicated unsatisfactory regulation of the metabolome. Taken together, HUA significantly altered the metabolic profile of plasma, urine, and faces in rat and different drug treatments exerted treatment effects at different degree.

**TABLE 1:** Pathway analysis of differential metabolites identified between control and HAU groups rats. Raw p is the original *p* value calculated from the enrichment analysis; the Holm p is the *p* value adjusted by Holm-Bonferroni method; the FDR p is the *p* value adjusted using False Discovery Rate.

	Metabolism pathways	Raw p	Holm adjust	FDR
Plasma	Fatty acid degradation	2.87E-03	1.15E-02	7.16E-03
	Steroid hormone biosynthesis	2.04E-02	3.50E-02	2.04E-02
	Pyrimidine metabolism	8.95E-05	4.48E-04	4.48E-04
	Glycerophospholipid metabolism	1.75E-02	3.50E-02	2.04E-02
	Tryptophan metabolism	6.06E-03	1.82E-02	1.01E-02
Feces	alpha-Linolenic acid metabolism	3.35E-02	1.34E-01	3.39E-02
	Biosynthesis of unsaturated fatty acids	3.35E-02	1.34E-01	3.39E-02
	Steroid hormone biosynthesis	4.26E-02	1.44E-01	4.39E-02
	Primary bile acid biosynthesis	5.39E-02	1.51E-01	4.78E-02
Urine	Phenylalanine metabolism	4.00E-04	1.16E-03	1.16E-03
	Riboflavin metabolism	2.50E-03	4.95E-03	3.71E-03
	Vitamin B6 metabolism	1.76E-02	1.76E-02	1.76E-02

### *Astragalus membranaceus* ultrafine powder administration reversed severe disruption of lipid metabolism caused by hyperuricemia

Lipid metabolism is an essential and complex physiological process involved in nutrient adjustment, hormone regulation, and homeostasis (Li et al., 2017). Lipid metabolism disorder results in phagocytosis of lipids by macrophages to form foam cells, which can accelerate atherosclerotic plaque formation (Inose et al., 2015). Lipids are digested into short-chain fatty acids, long-chain fatty acids (LCFA), glycerol, and other metabolites by various enzymes and bile salts. Acylcarnitine (AC) facilitates entry of long-chain fatty acids into mitochondria *via* the carnitine shuttle to ß-oxidation (Figure 5B) (Ramsay et al., 2001; Jones et al., 2010). Alteration of AC can induce mitochondrial dysfunction and has been associated with obesity, arrhythmias, cardiac ischemia, and insulin resistance. (Meierhofer 2019).

**FIGURE 2:**
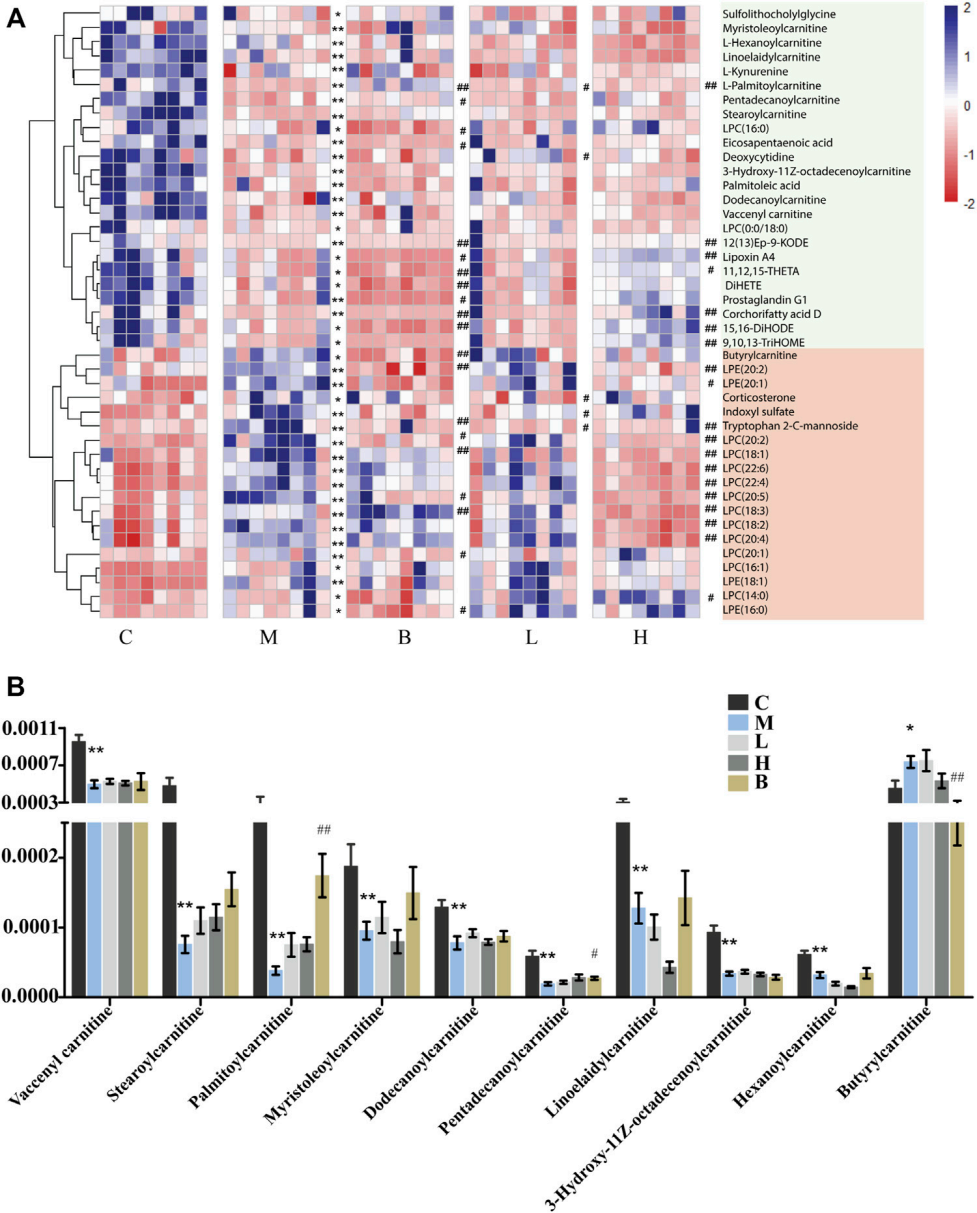
Differentially abundant metabolites in the plasma of HUA rats in control group (C), HUA model group (M), positive drug (benzbromarone) group (B), low-dose AMUP group (L), and high-dose AMUP group (H). **(A)** Heatmap of the differentially abundant metabolites in the plasma of HUA rats. Each cell represents one compound. Deeper blue represented higher levels and deeper red represented lower levels. **(B)** Comparison of signal intensity of significantly differentially abundant acylcarnitines in the five groups. *p < 0.05 compared with group C. **p < 0.01 compared with group C; #*p* < 0.05 compared with group M; ##*p* < 0.01 compared with group M.

In this study, we found that the levels of many LCFA esters of carnitine were dramatically altered in the plasma of the HUA rats (Figure 2A), including palmitoylcarnitine, myristoleoylcarnitine, stearoylcarnitine, vaccenyl carnitine, dodecanoylcarnitine, linoelaidylcarnitine, hexanoylcarnitine, 3-Hydroxy-11Z-octadecenoylcarnitine, pentadecanoylcarnitine, and butyrylcarnitine. As shown in Figure 2B, the levels of nine of 10 acylcarnitines in the plasma of the rats in the model group were significantly lower in the HUA group than those in the C group, which indicated that mitochondrial ß-oxidation of LCFAs in mitochondria was disrupted in the rats with HUA. Treatment with AM or benbromomalone resulted in a trend toward an increase in the levels of these metabolites compared with the levels observed in the HUA group (Figure 2B). Unlike other acylcarnitines, butyrylcarnitine can inhibit the activity of short-chain acyl-CoA dehydrogenase, which is the most vulnerable enzyme in the process of fatty acid ß-oxidation in mitochondria (Lopez-Suarez et al., 2019). Elevation of butyrylcarnitine in the blood of the HUA rats could result in disordered fatty acid oxidation (Wolfe et al., 1993). Treatment with high dose AMUP and benbromomalone decreased the levels of butyrylcarnitine. Inhibition of cellular LCFA oxidation could result in increased levels of triglycerides composed of LCFAs in the blood, leading to hyperlipidemia (Figure 5B). This may be a reason that 60% of patients with HUA suffer from hyperlipidemia (Guo et al., 2020).

**FIGURE 3:**
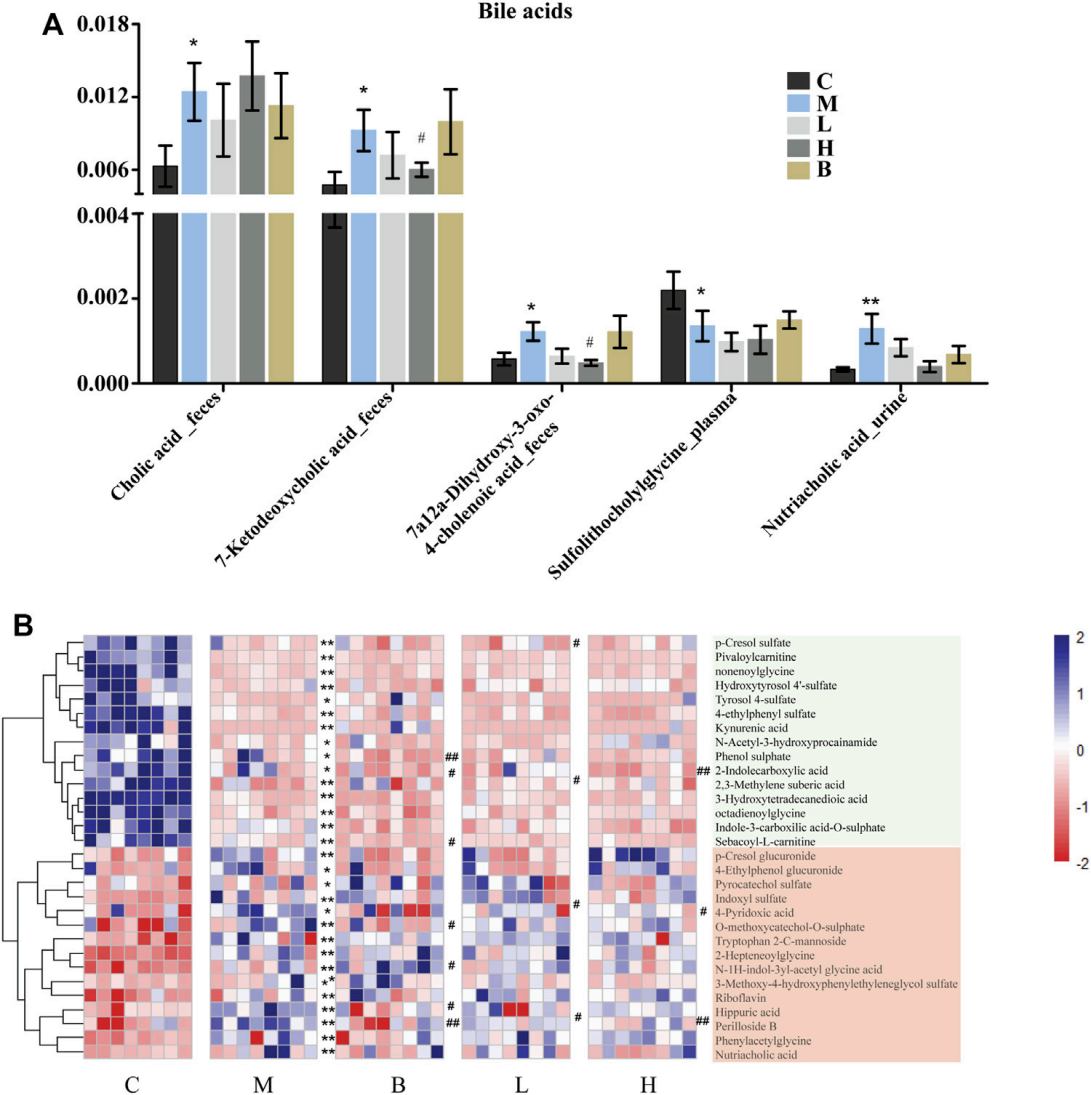
Significantly differentially abundant bile acid metabolites in HUA rats and the differentially abundant metabolites in the urine of HUA rats. **(A)** Comparison of signal intensity of significantly differentially abundant bile acid metabolites in control group (C), HUA model group (M), positive drug (benzbromarone) group (B), low-dose AMUP group (L), and high-dose AMUP group (H). **(B)** Heatmap of the differentially abundant metabolites in the urine of HUA rats. Each cell represents one compound. Deeper blue represents higher levels and deeper red represents lower levels. *p < 0.05 compared with group C; **p < 0.01 compared with group C; #*p* < 0.05 compared with group M; ##*p* < 0.01 compared with group M.

Bile acids are essential for absorption of dietary fats and vitamins, and have been implicated in regulation all the key enzymes involved in cholesterol homeostasis. Bile acids can be divided into two categories according to their structure: 1) free bile acids, such as cholic acid, deoxycholic acid, chenodeoxycholic acid, and lithocholic acid; and 2) conjugates of free bile acids with glycine or taurine, termed bound bile acids, such as cholic glycine acid, glycine chenodeoxycholic acid, taurocholic acid, and taurine chenodeoxycholic acid. Bile acids have potent toxic properties (e.g., membrane disruption) (St-Pierre et al., 2001; Davis et al., 2002; Chiang 2003; Claudel et al., 2005), and high levels of bile acids in the body can induce organ injury. The levels of the cholic acid and some of its intermediates, which contribute to lipid lipolysis and play essential roles in metabolic homeostasis and innate immunity (Liu and Wang 2019), were significantly altered in HUA rats. Cholic acid was proposed as a potential biomarker for liver injury (Smith et al., 2004). Furthermore, the levels of cholic acid were higher in the intestines of the rats in the model group than those in the control group (Figure 3A), which indicated that HUA could cause liver damage (Zhou et al., 2021). In addition, the levels 7-ketodeoxycholic acid and 7a,12a-dihydroxy-3-oxo-4-choleric acid, which are intermediates in the biotransformation of conjugates of cholic acid and chenodeoxycholic acid to unconjugated secondary bile acids (Sutherland et al., 1984; Song et al., 2019; Quinn et al., 2020), were also significantly higher in the intestines of the rats in the M group than those in the C group. The levels of cholic acid and chenodeoxycholic acid were restored to levels similar to those in the control group following treatment with AMUP, but not following treatment with benbromomalone. This result indicated that AMUP administration more effectively normalized bile acid metabolism than benbromomalone. In addition, the levels of sulfolithocholylglycine and nutriacholic acid were also significantly altered in HUA rats. Sulfolithocholylglycine is a secondary bile acid produced by enzymes in the microbial flora of the colonic environment (Goto et al., 2007). The levels of sulfolithocholylglycine were significantly reduced in the plasma of the HUA rats compared with those in the control group (Figure 3A), which indicated that the intestinal microbial community was significantly impacted by HUA. The levels of nutriacholic acid, a bile acid, were dramatically increased in the urine of the HUA rats compared to those in control rats, and high-dose AMUP showed a trend toward reversal of this decrease (Figure 3A). These results showed that HUA resulted in severely disordered bile acid metabolism and fatty acid degradation (Figure 5A), and treatment with AMUP significantly improved HUA-induced dysregulation of lipid metabolism.

### Lipid signaling molecules may be biomarkers of hyperuricemia

**FIGURE 4:**
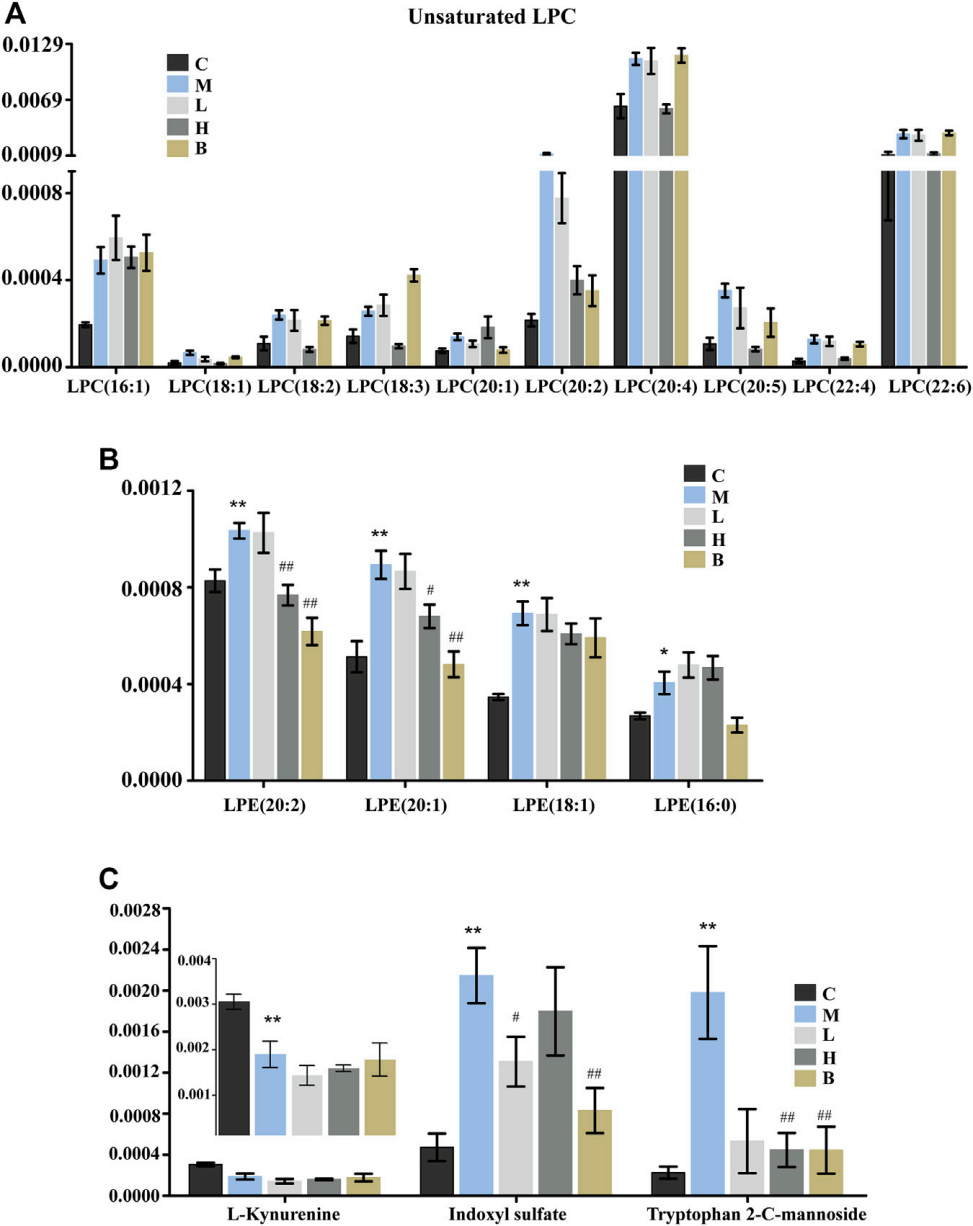
Significantly differentially abundant of lysophospholipids and tryptophan metabolites in HUA rats in control group (C), HUA model group (M), positive drug (benzbromarone) group (B), low-dose AMUP group (L), and high-dose AMUP group (H). **(A)** The levels of many unsaturated LPC were significantly higher in the blood of HUA rats than those in normal rats. **(B)** All LPE detected in the plasma were increased the model group. **(C)** Significantly differentially abundant metabolites of tryptophan in the plasma of the HUA rats. *p < 0.05 compared with group C; **p < 0.01 compared with group C; #*p* < 0.05 compared with group M; ##*p* < 0.01 compared with group M.

**FIGURE 5:**
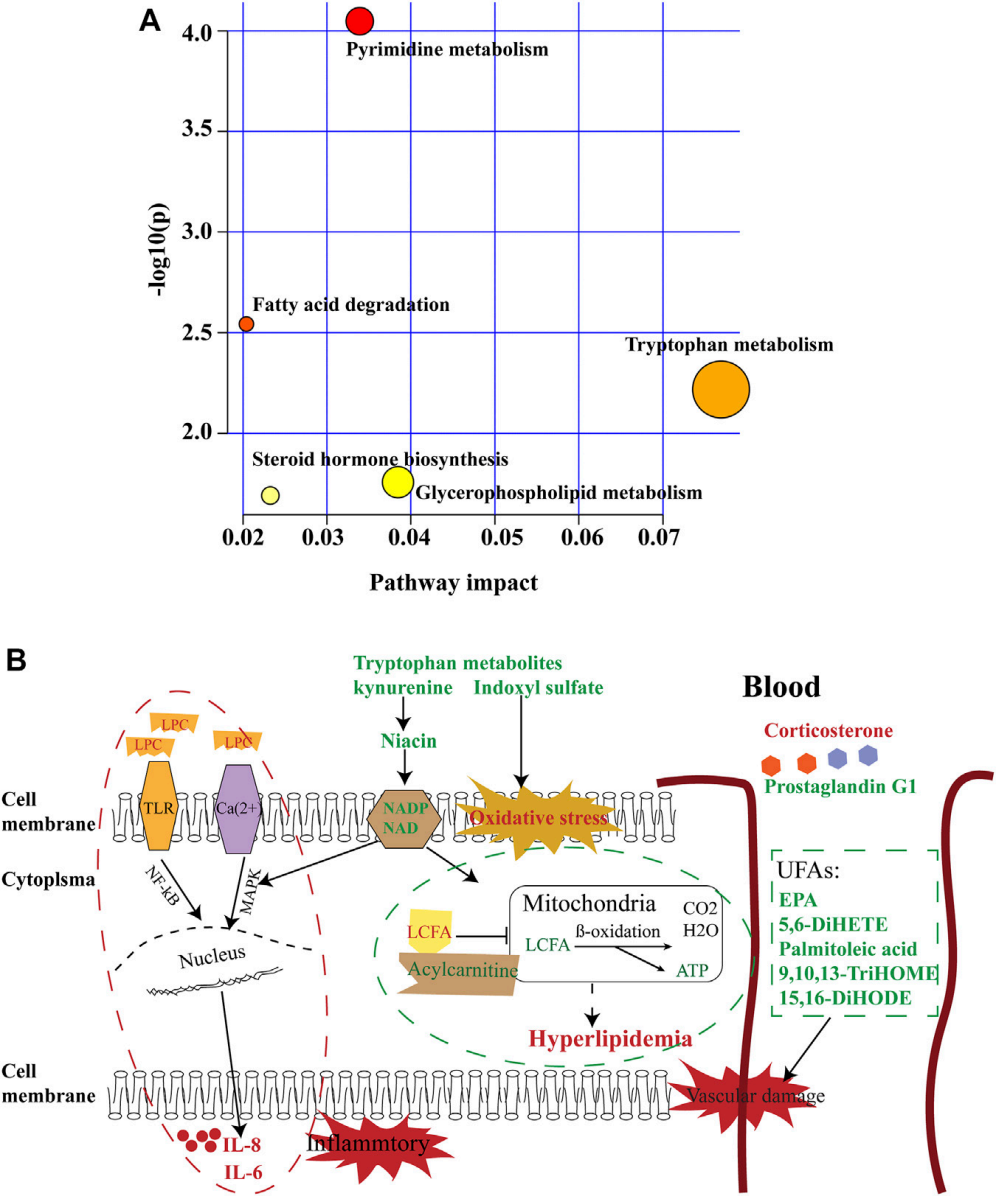
Summary of pathway analysis. **(A)** Pathways of metabolic abnormalities caused by HUA. Pathway impact value calculated from pathway topology analysis; a small *p*-value and large pathway impact factor indicate the pathway was significantly affected. **(B)** Diagram of abnormal metabolic pathways in rats with HUA. Red color indicates increased metabolites, green color indicates decreased metabolites.

Lysophospholipids (LPLs), which have hormone-like signaling properties, have largely been viewed only as membrane components necessary to mediate phospholipid synthesis and embed proteins into cell membranes (Fuchs et al., 2012). Lysophospholipids play important roles in physiological and pathophysiological processes, including smooth muscle contraction, proliferation, pain, inflammation, atherosclerosis, myocardial injury, and cancer cell migration and invasion. (Goetzl et al., 2002; Huang et al., 2002; Meyer zu Heringdorf and Jakobs 2007). Lysophosphatidylcholines (LPC) and lysophosphatidylethanolamines (LPE) belong to the LPL family. Lysophosphatidylcholine is a bioactive lysophospholipid produced by hydrolysis of phosphatidylcholine by phospholipase A2 (PLA2) in the Lands cycle (Law et al., 2019). Lysophosphatidylcholine can be categorized as short-chain LPC or saturated/unsaturated LPC according to acyl-chain length and degree of saturation. The functions of saturated and unsaturated LPCs differ significantly in disease states in different cells (Liu et al., 2020). Specifically, LPC has been shown to induce pro-inflammatory effects and vascular dysfunction in atherosclerosis and other cardiovascular diseases. (Liu et al., 2020). Levels of LPC can be increased by secretory PLA2-mediated metabolism or through oxidative modification of lipoprotein phospholipids under inflammatory conditions (Fuchs et al., 2005). We found that the levels of many unsaturated LPC (Figure 4A), including 18:1, 18:2, 18:3, 20:2, 20:4, 20:5, 22:4, and 22:6, which promote release of inflammatory cytokines (IL-6 and IL-8) and induce cyclooxygenase-2 expression (Figure 5B) (Yoon et al., 2012), were significantly elevated in the blood of HUA rats compared to those in the C group. These results suggested that patients with HUA who experience increased inflammation may be at higher risk for development of cardiovascular diseases (Zhang et al., 2019). However, except for LPC (20:1, 18:3), AM reversed the HUA-induced changes in unsaturated LPC levels to a greater degree than benbromomalone, which suggested that AM may provide better protection against LPC-induced dysfunction (Figure 4A). In addition, abnormal LPC levels have been shown to induce inflammation and apoptosis of neurocytes and promote demyelination, which may promote development of Alzheimer’s disease (AD) (Ikeno et al., 2009). We found that AMUP administration restored LPC to levels similar to those observed in the control group, which suggested that AM may also be a candidate for prevention of development of AD. In contrast to LPC, all LPE (LPE 16:0, 18:1, 20:2, 20:1) species detected in the plasma were increased in the model group (Figure 4B). Increased levels of LPEs have been associated with many diseases, including hyperlipidemia and atherosclerosis (Zhao et al., 2018). These results indicated that LPC and LPE may be potential blood biomarkers for screening of patients with HUA.

### Hyperuricemia-induced tryptophan metabolism disorder was resistant to drug treatment

Tryptophan is an essential amino acid. Tryptophan metabolism within different tissues is associated with numerous physiological functions (Le Floc’h et al., 2011). Levels of several metabolites of tryptophan, including kynurenine, indoxyl sulfate, and tryptophan 2-C-mannoside, were significantly altered in HUA rats, which indicated that tryptophan metabolism was disrupted in HUA rats (Figure 5A). Kynurenine is used to produce niacin. Niacin (also known as “vitamin B3” or “vitamin PP”) includes two vitamers (nicotinic acid and nicotinamide), which are precursors to the coenzyme nicotinamide adenine dinucleotide (NAD) and nicotinamide adenine dinucleotide phosphate (NADP). These coenzymes are required for oxidative reactions crucial for energy production. In addition, they are also substrates for enzymes involved in non-redox signaling pathways (NF-κB and MAPK), and contribute to regulation of biological functions such as gene expression, cell cycle progression, DNA repair, and cell death (Figure 5B). Vitamin B3 has long been recognized as a critical mediator of neuronal development and survival in the central nervous system. (Gasperi et al., 2019). The degradation of the kynurenine has been observed in stroke, epilepsy, multiple sclerosis, amyotrophic lateral sclerosis, Parkinson’s disease, Huntington’s disease, and Alzheimer’s disease. (Erhardt et al., 2017; Schwarcz and Stone 2017; Platten et al., 2019). In this study, the levels of kynurenine in the plasma and urine of the HUA rats were significantly lower than those in normal rats (Figures 3B, 4C and Supplementary Table S1), and neither AM nor benbromomalone treatment reversed this decrease, which suggests drugs hardly restored tryptophan-kynurenine metabolism disorder caused by HUA. Indoxyl sulfate is a circulating uremic toxin that induces glomerular sclerosis and interstitial fibrosis (Duranton et al., 2012) and increases the rate of progression of renal failure. In plasma, indoxyl sulfate is a protein-bound uremic solute that induces endothelial dysfunction by inhibiting endothelial proliferation and migration *in vitro* (Brunet et al., 2003). Indoxyl sulfate can increases reactive oxygen species (ROS) production in tubular cells and increase NAD(P)H oxidase activity in endothelial cells (Figure 5B) (Dou et al., 2007). In our study, indoxyl sulfate levels were significantly increased in the plasma and urine of the rats with HUA compared with those in the C group (Figures 3B, 4C and Supplementary Table S1). This increase was reversed following treatment with the AM or benbromomalone, which indicated that AM could protect against endothelial cell damage caused by increased indoxyl sulfate (Figure 4C). A previous study showed that tryptophan 2-C-mannoside level was significantly higher in individuals with kidney disease than that in individuals without chronic kidney disease (Sekula et al., 2017). The levels of tryptophan 2-C-mannoside were elevated in the plasma and urine of HUA rats compared to those in rats in C group, which indicated that HUA could cause chronic kidney damage. HUA-induced increases in tryptophan 2-C-mannoside in plasma, but not in urine, were reversed by treatment with AM or benbromomalone (Figure 4C). Since tryptophan 2-C-mannoside cannot be catabolized inside the body, but rather excreted in urine (Minakata et al., 2021), these results suggest that the drug treatment can partially restore kidney function and excrete excess tryptophan 2-C-mannoside *via* urine. Besides, autophagy can upregulate the level of tryptophan 2-C-mannoside in cultured cells (Minakata et al., 2020), the still elevated tryptophan 2-C-mannoside in the urine may indicate HUA induced cell death may still ongoing even after drug treatment.

### Hyperuricemia-induced changes in steroid biosynthesis were reversed by *Astragalus membranaceus* ultrafine powder

Hormones regulate many bodily functions, including growth and development, metabolism, electrolyte balances, and reproduction (Hiller-Sturmhofel and Bartke 1998). The levels of corticosterone, dihydrocortisol, and prostaglandin G1 were significantly altered in HUA rats. Pathway analysis showed that steroid hormone biosynthesis was disrupted in HUA rats (Figure 5A). Corticosterone and dihydrocortisol secreted by the adrenal gland play a crucial role in many biological processes. Corticosterone is the precursor molecule to the mineralocorticoid aldosterone, a major homeostatic modulator of sodium and potassium levels *in vivo* (Ghulam et al., 1999). In many species, including amphibians, reptiles, rodents, and birds, corticosterone is the main glucocorticoid and is involved in energy regulation, immune reactions, and stress responses (Oprica et al., 2005). The metabolic actions of corticosterone are strictly dose-dependent (Devenport et al., 1989). Dihydrocortisol is a glucocorticoid secreted by the adrenal gland that plays a role in regulating the hydro-salinity balance, glucose metabolism, and growth and development (Mikami et al., 1980; Kallubai et al., 2019). In addition, dihydrocortisol has anti-inflammatory properties and suppresses the immune response (Southren et al., 1985). The levels of corticosterone and dihydrocortisol were significantly increased in the plasma and feces of the HUA rats, which suggested that HUA may damage the adrenal gland and may induce adrenal cortical hyperplasia. Treatment with low-dose of AM reversed the HUA-induced changes in corticosterone and dihydrocortisol (Figure 2A and Supplementary Table S1). Prostaglandin G1 is a small molecular pro-inflammatory mediator derived from arachidonic acid that plays roles in, inflammation, pain modulation, allergies, and bone formation (Doucette and Walter 2017). An absolute or relative deficiency of prostaglandins has been observed in many diseases (Moran and Nicholson 1990). The levels of prostaglandin G1in the plasma of HUA rats was significantly lower than those in the C, L, and H groups (Figure 2A). Treatment with benbromomalone further reduced the levels of prostaglandin G1 in the blood of HUA rats, which suggested that benbromomalone could exacerbate HUA-related dysfunction.

### Malabsorption of unsaturated fatty acids in hyperuricemia rats

The health benefits of unsaturated fatty acids (UFAs) have been well-established. Specifically, UFAs have been shown to exert cardioprotective, anti-inflammatory, anticancer, and antimicrobial effects, and can positively influence skin conditions (Bang et al., 1980; Tvrzicka et al., 2011). Some unsaturated fatty acids cannot be synthesized by the human body and need to be supplied in the diet food. These essential fats include linoleic acid, linolenic acid, and arachidonic acid. Unsaturated fatty acids are absorbed by small intestinal epithelial cells and incorporated into high-density lipoprotein, which has anti-atherosclerotic properties. The levels of eicosapentaenoic acid (EPA), palmitoleic acid, linoleic acid and their metabolites were significantly altered in HUA rates compared with those in normal rats (Figure 2A). Eicosapentaenoic acid is an essential polyunsaturated fatty that serves as the precursor for the prostaglandin-3 and thromboxane-3 families, and inhibits arachidonic acid conversion into the thromboxane-2 and prostaglandin-2 families to prevent thrombosis (Nelson and Raskin 2019). Eicosapentaenoic acid and its metabolites exert anti-inflammatory effects and reduce the incidence of cardiovascular disorders in humans and experimental animals (Kida et al., 2020). We found that the levels of EPA and the EPA-derived anti-inflammatory lipid mediator 5,6-DiHETE (Hamabata et al., 2018) were significantly decreased in the plasma of the model group rats compared to those in normal rats (Figure 2A). Furthermore, the levels of EPA and 5,6-DiHETE in the plasma of the rats in the B group were lower than those in HUA rats (Figure 2A), which indicated that treatment with benbromomalone may result in increased risk of cardiovascular disease and inflammation. In contrast, treatment with EPA increased the levels of EPA and 5,6-DiHETE in the plasma of HUA rats, which indicated that AM could decrease inflammation and prevent cardiovascular disease in HUA rats. Palmitoleic acid, an omega-7 monounsaturated fatty acid, is an abundant component of glycerides on human adipose tissue. Palmitoleic acid levels were significantly decreased in the blood of HUA rats compared with those in control rats. This decrease was partially reversed by AM, but not by benbromomalone. In addition, the levels of three linoleic acid-derived lipid mediators, 9,10,13-TriHOME, 15,16-DiHODE, and corchorifatty acid D were significantly lower in the plasma of the HUA rats than those in control rats (Figure 2A). Decreased 9,10,13-TriHOME and 15,16-DiHODE may promote inflammation (Fuchs et al., 2018; Tan et al., 2019), which could contribute to a more robust inflammatory response in HUA rats. Corchorifatty acids A, B, and C have been shown to inhibit lipopolysaccharide-induced nitric oxide production in cultured mouse peritoneal macrophages (Yoshikawa et al., 1998). The function of corchorifatty acid D has not been characterized. Treatment with high-dose AMUP reversed the HUA-induced decreases in the levels of 9,10,13-TriHOME, 15,16-DiHODE, and corchorifatty acid D. The levels of the polyunsaturated fatty acid-derived diol metabolite 19,20-diHDPA, which exerts anti-inflammatory effects (Heemskerk et al., 2014), were decreased in the feces of HUA rats (Supplementary Table S1). Changes in levels of unsaturated fatty acids indicated that HUA rats may suffer from poor absorption of UFAs and may be at increased risk for development of inflammatory and cardiovascular diseases (Figure 5B). Administration of AMUP may exert greater therapeutic effects than benbromomalone against HUA by altering levels of unsaturated fatty acids. Proper dietary supplementation with UFAs may also alleviate HUA.

### Changes in inflammatory-associated metabolites indicated that chronic inflammation occurred in hyperuricemia rats

11,12,15-THETA, which activates K^+^ channels to hyperpolarize the aortic smooth muscle membrane and induce relaxation, is produced from arachidonic acid by endothelial cells. (Campbell et al., 2003; Chawengsub et al., 2009). Decreased ability to relax the aortic smooth muscle membranes could contribute to depressed vascular reactivity (Rinaldi 2005), leading to cardiovascular disease. We found that the levels of 11,12,15-THETA in the plasma of rats in the M and B groups were significantly lower than those in the C, L, and H groups (Figure 2A), which indicated AM, but not benbromomalone, significantly reversed HUA-induced changes in relaxation ability of aortic smooth muscle membranes. Therefore, AMUP administration reduced the risk of cardiovascular disease in rats with HUA. Lipoxin A4 (LXA4) is a product of 5-lipoxygenase activity in activated leukocytes. Lipoxins (LXs) are generated from arachidonic acid *via* sequential actions of lipoxygenases. Lipoxin A4 exerts potent anti-inflammatory and pro-resolving effects *in vivo* in some animal model systems (Serhan 2005; Parkinson 2006). Neutrophils can kill pathogens and are the most abundant type of white blood cell in the peripheral blood. 3,4-Methylenesebacic acid has been shown to be a biomarker of neutropenia (Deng et al., 2020). We found that LXA4 and 3,4-methylenesebacic acid levels were lower in the plasma and feces of rats with HUA (Supplementary Table S1), which suggested that HUA rats had impaired anti-inflammatory capacity. Administration of high dose AM, but not low dose AM or benbromomalone, returned the levels of LXA4 to those observed in normal rats, which suggested that high dose AM could exert anti-inflammatory effects through increased levels of LXA4. Lower LXA4 levels in the B group indicated that treatment of HUA with benbromomalone may weaken the host immune response. These results suggested that inflammatory factors were increased in rats with HUA, and AMUP treatment could reduce this inflammation.

## Conclusion

Metabolic changes are the direct results of alterations in protein and enzyme activities. Therefore, metabolomics may offer valuable information on HUA-related physiological processes, molecular interactions, and metabolic pathways. By providing an overall “fingerprint” of metabolite alterations in multiple biofluids, multi-metabolomics analysis has identified many potential biomarkers and therapeutic targets. This study found that the levels of many metabolites were significantly altered in rats with HUA compared to those in normal rats, and changes in metabolite levels in urine were resistant to treatment with benbromomalone or AMUP. In addition, lipid metabolism, lipid signaling molecules, hormones synthesis, UFA absorption, and tryptophan metabolism were altered in HUA rats, which could contribute to hyperlipidemia, cardiovascular and cerebrovascular diseases, chronic inflammation, or Alzheimer’s disease. However, the levels of metabolites in these pathways returned to normal following treatment with benbromomalone and AM. AM exerted better therapeutic effects on some pathways than benbromomalone, such as bile acid metabolism, hormones synthesis, and UFA absorption. Therefore, AMUP has great potential for the treatment of HUA. In addition, additional supplementation of UFAs and tryptophan may alleviate hyperuricemia.

## Data Availability

The original contributions presented in the study are included in the article/Supplementary Material, further inquiries can be directed to the corresponding author.
